# Maintenance Therapy with Trastuzumab in Her2 Positive Metastatic Parotid Ductal Adenocarcinoma

**DOI:** 10.1155/2014/162534

**Published:** 2014-07-17

**Authors:** Muhammad Shahid Iqbal, Ghazia Shaikh, Sanjoy Chatterjee, Helen Cocks, Josef Kovarik

**Affiliations:** ^1^Department of Clinical Oncology, Northern Centre for Cancer Care, Freeman Hospital, Newcastle upon Tyne NE7 7DN, UK; ^2^Department of Oncology, Tata Medical Centre, Kolkata 700 156, India; ^3^Department of Otolaryngology, Sunderland Royal Hospital, Kayll Road, Sunderland SR4 7TP, UK

## Abstract

Salivary ductal carcinomas (SDCs) are extremely rare and aggressive malignancies, accounting for approximately 6% of all salivary gland malignancies. One distinct feature is their resemblance to ductal carcinomas of breast. A significant percentage of SDCs overexpress Her2 and the use of targeted therapy with trastuzumab can be considered in these patients. We report a rare case of long term disease control with trastuzumab in Her2 positive metastatic parotid ductal carcinoma. Our case also highlights that isolated brain metastasis should be managed aggressively to allow optimal local control when systemic disease is under remission with trastuzumab. We have also reviewed the published literature on the use of trastuzumab in SDCs.

## 1. Case History

A 59-year-old gentleman presented with six-week history of a rapid increase in size of a previously asymptomatic right sided parotid swelling which had been present for 15 years. Examination showed a right parotid swelling which was painful and an associated partial right facial palsy, House-Brackmann grade III [1]. Fine needle aspiration showed malignant cells and computed tomography showed a large parotid mass with enlarged lymph nodes at levels Ib and II but no distant metastases. The patient underwent a right total parotidectomy with facial nerve resection and neck dissection levels I–V. Pathology revealed a poorly differentiated ductal adenocarcinoma of parotid gland, measuring 40 mm in size, 0.3 mm clear of the resection margin. Two out of five lymph nodes at level I, six out of twelve at level II, all four at level III, one out of four at level IV, and all sixteen at level V contained metastatic disease. There was evidence of extra capsular spread. The section of facial nerve was also infiltrated. The tumour was staged as pT4a pN2b M0 [2]. The tumour cells showed strong and uniform labeling for human epidermal growth factor receptor, Her2/neu, 3+ by immunohistochemistry (IHC) and the presence of Her2 gene amplification was confirmed by fluorescence in situ hybridization (FISH). The patient received adjuvant radiotherapy to the parotid tumour bed and right neck to a dose of 63 Gy in 30 fractions over a period of 41 days. He tolerated radiotherapy fairly well.

Six months later, the patient developed backache and his GP requested a plain X-ray which suggested metastatic disease. Magnetic resonance imaging (MRI) revealed multiple metastatic deposits throughout the spine and sacrum with incipient spinal cord compression at the level of third thoracic vertebra, T3. Biopsy of the spinal metastases confirmed metastatic ductal carcinoma of parotid gland and Her2 was overexpressed. He received urgent palliative radiotherapy to a dose of 20 Gy in 5 fractions at levels T2–T5 and T12–L5 and he started palliative chemotherapy with docetexal on a 3-weekly basis (first 3 cycles with 75 mg/m^2^ and as he tolerated it well, the dose was escalated to 100 mg/m^2^) along with trastuzumab (loading dose of 8 mg/kg followed by 6 mg/kg on a 3-weekly basis) since the tumour was Her2 positive. He completed 6 cycles of docetaxel without reporting any significant toxicity whilst trastuzumab was continued as maintenance. MR and CT scan showed a partial response to the treatment.

**Figure 1:**
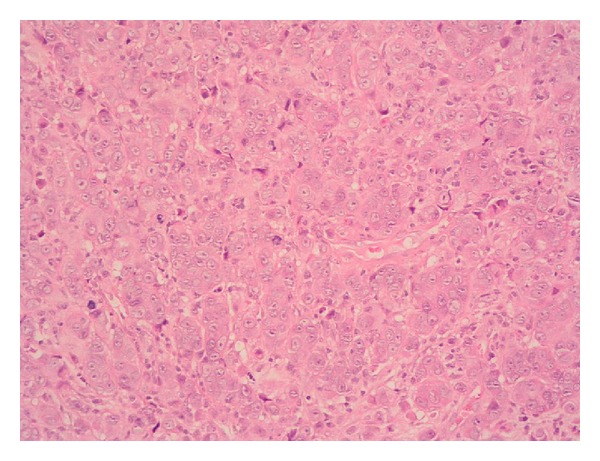
Hematoxylin and eosin stained section of cerebellar lesion at 20x magnification showing general cytoarchitectural features of the tumour.

**Figure 2:**
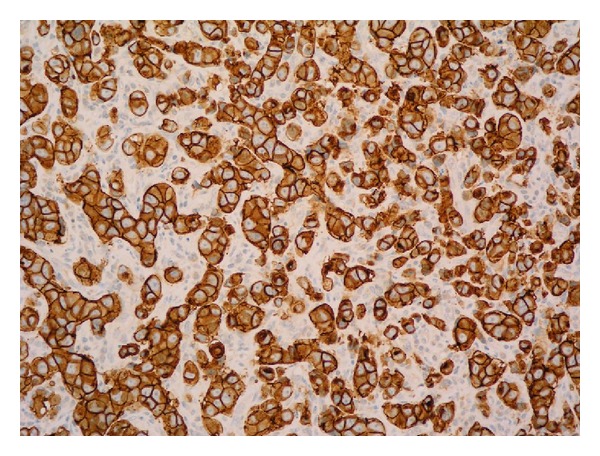
HER2 immunohistochemistry of cerebellar lesion—the vast majority of the neoplastic cells are positive with strong cytoplasmic membrane staining.

A further 8 months later, he presented with increased lethargy, vomiting, weakness, and incoordination. An MRI of spine showed extensive bony metastases but no sign of further cord compression. A CT head however showed a solitary left cerebellar mass obstructing the cerebral aqueduct. He underwent a posterior fossa craniotomy and excision of the metastasis. The histology confirmed metastatic parotid gland ductal carcinoma and Her2 was overexpressed (Figures 1 and 2). Postoperatively he received 20 Gy in 5 fractions of adjuvant radiotherapy to the whole brain. Trastuzumab was discontinued at the time of craniotomy.

Six months later, an MRI scan of brain showed stable appearances. Although he had developed a brain metastasis whilst on trastuzumab, maintenance therapy with trastuzumab was restarted since there was no evidence of progressive disease (following an eight-month gap). At the time of writing, the patient is alive, more than 5 years since his diagnosis, and has received forty-two months of trastuzumab maintenance therapy without interruption. He remains well with no new symptoms and his disease remains stable radiologically.

## 2. Discussion

Salivary gland cancers are rare, accounting for less than 5% of all cancers of the head and neck. Salivary duct carcinomas (SDCs) are extremely rare, accounting for approximately 6% of all salivary gland malignancies [3, 4]. The vast majority of these (85%) originate in the parotid gland [5]. The salient features of SDCs are presentation at advanced stage, facial nerve invasion, and increased potential of regional lymph nodal involvement and distant metastases. Another distinct feature is its histological resemblance with ductal carcinoma of breast [6]. This histological similarity has led to the study of human epidermal growth factor receptor Her2/neu overexpression in SDCs. Her2 is amplified in approximately 20% of the invasive breast cancers and independently is considered a worse prognostic factor [7]. Trastuzumab, a recombinant humanised monoclonal antibody, binds the extracellular domain of Her2 with high affinity and thus inhibits proliferation of tumour cells that overexpress Her2. It is a well-recognised therapy in breast and gastric tumours that overexpress Her2 [8, 9].

Primary surgery is the standard treatment for nonmetastatic operable SDCs with radiotherapy used in the adjuvant setting to improve local control in high risk disease. Systemic chemotherapy is reserved for unresectable locoregional recurrence or where there are distant metastases for palliative management. Various regimes of single agent or combination chemotherapy have been assessed in small sample size studies with a 15–50% response rate for a duration of 6–9 months [10].

It has been reported that the percentage of Her2 overexpression in SDCs is approximately 37% as assessed by IHC and 72% as determined by FISH [11] and there is evidence that this overexpression is associated with an aggressive behaviour [12].

The evidence for optimal management for these aggressive malignancies is lacking mainly due to the rarity of the disease. Several case reports and three small case series have been published using trastuzumab alone or in combination with other agents (see Table 1). The follow-up period varies. Only one study used trastuzumab alone as a part of phase II trial and only one of 14 patients showed a partial response [13]. Lapatinib, an orally active tyrosine kinase inhibitor which inhibits both Her2 and EGFR receptors, has also been evaluated in a phase II study but no objective response was observed [14]. Although our patient initially received six cycles of docetaxel in combination with trastuzumab, he remained on trastuzumab alone for the last 40 months and remained well with radiologically stable disease.

Interestingly, our patient is one of the first reported cases in the literature, having a documented brain metastasis on maintenance trastuzumab for SDC. This is possibly secondary to a lower bioavailability of trastuzumab due to the blood brain barrier. Aggressive management with surgery followed by radiation therapy has allowed long term control of the disease. This case highlights the concept of “sanctuary site” metastasis often seen in Her2 positive breast cancer patients [15]. Lapatinib is often prescribed for brain metastasis in Her2 positive breast cancer after progression on trastuzumab because of its higher bioavailability in the brain [16]. Lapatinib may therefore be explored if SDC patients develop progressive brain only metastasis in due course.

## 3. Conclusion

SDCs are highly aggressive malignant tumours. Given the rarity of the disease, the evidence for optimal management is lacking. However, given the histological resemblance with ductal carcinoma of breast, targeted therapy with trastuzumab targeting Her2 overexpression is a reasonable option in recurrent/metastatic setting and it should be considered on individual basis.

## Figures and Tables

**Table 1:** Summary of use of trastuzumab in Her2 overexpressed metastatic SDCs.

Study ID	Method	*n*	Chemotherapy used concurrently	Response	Outcome
Haddad et al., 2003 [13]	Phase II trial	14	No	1/14 PR	Median time to progression 4.2 months
Limaye et al., 2013 [17]	Retrospective case series	5∗	Carboplatin/paclitaxel	1/5 CR 2/5 PR	Median duration of response 18 months (range: 8–52 months)
Kadowaki et al., 2013 [18]	Case report	1	Paclitaxel	CR	13-month f/u. No disease progression
Firwana et al., 2012 [19]	Case report	1	Paclitaxel	CR (of liver metastases)	Alive. Stable after 16 months of f/u
Nashed and Casasola, 2009 [20]	Case report	1	Initially doxorubicin then docetaxel	PR∗∗	Alive. 20 months since metastatic disease
Prat et al., 2008 [21]	Case report	1	Carboplatin/palclitaxel	CR	Alive. No disease progression in 14 months f/u
Sharon et al., 2010 [22]	Case report	1	Capecitabine/zoledronic acid	CR	Alive. No disease progression in 2-year f/u
Kaidar-Person et al., 2012 [23]	Case report	1	Carboplatin/paclitaxel	CR	Alive. Duration unrecorded
Locati et al., 2005 [24]	Cases series	4	Yes (not specified)	1/4 SD	Median time to progression 2.5 months

*n*: number of patients. PR: partial response. CR: complete response. SD: stable disease. f/u: follow-up.

∗Out of total 13 patients, 8 were treated adjuvantly and 5 were metastatic. ∗∗CR in lung and liver and minimal residual disease in neck.
